# HIV-1 tat expression and sulphamethoxazole hydroxylamine mediated oxidative stress alter the disulfide proteome in Jurkat T cells

**DOI:** 10.1186/s12985-018-0991-x

**Published:** 2018-05-09

**Authors:** Kemi Adeyanju, John R. Bend, Michael J. Rieder, Gregory A. Dekaban

**Affiliations:** 1 BioTherapeutics Research Laboratory, Molecular Medicine Research Laboratories, Robarts Research Institute, Rm 2214, 1151 Richmond Street North, London, Ontario Canada; 2 Department of Microbiology and Immunology, University of Western Ontario, 1151 Richmond Street North, London, ON N6A 5B7 Canada; 3 Department of Pathology and Laboratory Medicine, University of Western Ontario, 1151 Richmond Street North, London, ON N6A 5B7 Canada; 4 Drug Safety Laboratory, Molecular Medicine Research Laboratories, Robarts Research Institute, Rm 2214, 1151 Richmond Street North, London, Ontario Canada; 5 Department of Pediatrics, University of Western Ontario, 1151 Richmond Street North, London, ON N6A 5B7 Canada

**Keywords:** HIV, Adverse drug reaction, Sulphamethoxazole, Thiol proteome, Cysteine thiols, Peroxiredoxin

## Abstract

**Background:**

Adverse drug reactions (ADRs) are a significant problem for HIV patients, with the risk of developing ADRs increasing as the infection progresses to AIDS. However, the pathophysiology underlying ADRs remains unknown. Sulphamethoxazole (SMX) via its active metabolite SMX-hydroxlyamine, when used prophylactically for pneumocystis pneumonia in HIV-positive individuals, is responsible for a high incidence of ADRs. We previously demonstrated that the HIV infection and, more specifically, that the HIV-1 Tat protein can exacerbate SMX-HA-mediated ADRs. In the current study, Jurkat T cell lines expressing Tat and its deletion mutants were used to determine the effect of Tat on the thiol proteome in the presence and absence of SMX-HA revealing drug-dependent changes in the disulfide proteome in HIV infected cells.

Protein lysates from HIV infected Jurkat T cells and Jurkat T cells stably transfected with HIV Tat and Tat deletion mutants were subjected to quantitative slot blot analysis, western blot analysis and redox 2 dimensional (2D) gel electrophoresis to analyze the effects of SMX-HA on the thiol proteome.

**Results:**

Redox 2D gel electrophoresis demonstrated that untreated, Tat-expressing cells contain a number of proteins with oxidized thiols. The most prominent of these protein thiols was identified as peroxiredoxin. The untreated, Tat-expressing cell lines had lower levels of peroxiredoxin compared to the parental Jurkat E6.1 T cell line. Conversely, incubation with SMX-HA led to a 2- to 3-fold increase in thiol protein oxidation as well as a significant reduction in the level of peroxiredoxin in all the cell lines, particularly in the Tat-expressing cell lines.

**Conclusion:**

SMX-HA is an oxidant capable of inducing the oxidation of reactive protein cysteine thiols, the majority of which formed intermolecular protein bonds. The HIV Tat-expressing cell lines showed greater levels of oxidative stress than the Jurkat E6.1 cell line when treated with SMX-HA. Therefore, the combination of HIV Tat and SMX-HA appears to alter the activity of cellular proteins required for redox homeostasis and thereby accentuate the cytopathic effects associated with HIV infection of T cells that sets the stage for the initiation of an ADR.

**Electronic supplementary material:**

The online version of this article (10.1186/s12985-018-0991-x) contains supplementary material, which is available to authorized users.

## Background

Oxidative stress represents an imbalance in cellular homeostasis due to either the excessive production of reactive oxygen species (ROS), the impairment of cellular anti-oxidant defenses or both, which can lead to a disruption of redox control and signaling that can result in molecular damage [1]. During HIV infection, oxidative stress contributes to the impaired responsiveness, apoptosis and depletion of CD4+ T cells [2–6]. In addition, there is a strong association between decreased survival of HIV-infected individuals and low thiol levels [2, 7, 8]. This oxidative stress is primarily due to the HIV-1 transactivator of transcription (Tat) [9].

Tat influences the cellular redox state by two mechanisms; by depleting antioxidant concentrations and/or increasing oxidant levels. The capacity of Tat to decrease antioxidant concentrations is linked to the suppression of manganese superoxide dismutase (Mn-SOD) expression as well as its ability to directly decrease cellular glutathione (GSH) content by down-regulating glutathione synthetase [10–12]. In addition, Tat can induce ROS production in multiple cell types [13–15].

Tat is encoded by two exons and synthesized at early and late stages of HIV-1 replication [16]. However, during the viral life cycle, two different forms of Tat are generated. One is the product of two splicing events of the HIV-1 transcript that produces a full-length protein of 101 amino acids [17]. The second form encoded by only the first exon is 72 amino acids in length. In vitro, both variants efficiently transactivate HIV-1-specific transcription [18, 19]. Tat also contains a protein transduction domain (PTD) which enables the protein to be secreted from HIV-1-infected cells and enter uninfected cells to regulate host gene expression [16, 20]. The PTD also serves as a nuclear localization sequence (NLS) that restricts Tat to the nucleus preventing Tat from interacting with cellular protein that can result in cytopathic effects as we have recently demonstrated [21].

As HIV infection progresses to AIDS, there is a dramatic increase in the incidence of hypersensitivity adverse drug reactions (ADRs) [22]. These ADRs arise from the treatments involving all antiretroviral drug classes as well as medications used to treat opportunistic infections that arise during HIV disease progression [22]. The drug reported to produce the highest incidence of HIV-associated ADRs is Sulphamethoxazole (SMX), a sulphonamide used to prevent and treat pneumocystis pneumonia, a common opportunistic infection in individuals with advanced AIDS [23].

The major route of metabolism for SMX is N-acetylation in the liver, a detoxication reaction that results in a product rapidly eliminated from the body [24, 25]. A small and patient-variable fraction of a given dose of SMX is also oxidized by the P450 monooxygenase isozyme CYP2C9 to the reactive metabolite Sulphamethoxazole-hydroxylamine (SMX-HA). Further auto-oxidation gives rise to Sulphamethoxazole-nitroso (SMX-NO), an electrophile that can react covalently with cellular proteins forming haptens [26]. SMX-HA, and subsequently SMX-NO, can also be generated by dendritic cells and by myeloperoxidase (MPO) in activated neutrophils, monocyte/macrophages and lymphocytes [24, 25, 27, 28].

The mechanism(s) that lead(s) to the development of SMX-mediated ADRs is incompletely understood, however. There is evidence that in the absence of adequate detoxication, electrophilic SMX-NO preferentially react with reactive cysteine thiols of cellular proteins to form hapten-protein conjugates. Ionized protein cysteines are stronger nucleophiles than unionized protein thiols and serve as a very common site for covalent reaction with drug electrophiles [29]. Such drug hapten-protein conjugates can form neo-antigens that are recognized by the immune system [25, 30]. Upon processing and presentation of the hapten-protein conjugate, immune responses can be directed against all or part of the SMX-protein conjugate, which clinically manifests as a hypersensitivity ADR. Thus, the oxidative stress inherent during an HIV infection that results in reduced concentrations of the protective antioxidant GSH [2, 31, 32] and the formation of SMX-hapten-protein conjugates creates an environment favoring the development of ADRs in HIV^+^ patient populations that are progressing to AIDS.

Low levels of ROS cause reversible post-translational modifications of proteins and function as second messengers in signal transduction pathways to regulate multiple biological processes [33]. With increasing ROS concentration, the sulfur-containing residues, cysteine and methionine, are susceptible to oxidation and undergo reversible and non-reversible reduction–oxidation (redox) reactions [34, 35]. The oxidation and reduction of these ionized (reactive) cysteine residues primarily forms the basis of redox signalling [33]. The oxidation of reactive cysteine residues leads to formation of disulfide bonds (Cys-S-S-Cys) and cysteine-sulfenic (Cys-SOH), −sulfinic (Cys-SO_2_H) and -sulfonic (Cys-SO_3_H) acids [33, 34]. Reactive (ionized) cysteine thiol residues in proteins also react with GSH (S-glutathionylation) to form protein-glutathione mixed disulfides (PSSG) or to cysteine residues in other proteins to form protein-protein disulfides (PSSP) when GSH is depleted [34–36]. Importantly, Tat expression not only alters the overall pattern of gene expression within the infected cell, but also decreases intracellular GSH [9, 10, 12] and therefore may alter the disulfide proteome resulting in a cellular environment more susceptible to oxidative damage, cell death and ultimately to hypersensitivity ADRs.

The transactivation activity of Tat on HIV LTR-initiated RNA transcription is redox sensitive. The HIV Tat protein contains 7 cysteines that each form one or more disulfide bonds that are required for maximal Tat transactivator function [37, 38]. Protein disulfide formation is favored in a pro-oxidative environment. Under normoxic conditions, Tat functions optimally with respect to LTR-initiated transcription. However, in anoxic conditions or upon exposure to strong reducing agents Tat function is reduced or abolished, respectively [37, 38]. Thus, depending on the anatomical location of HIV infected T- and myeloid cells, Tat activity will, in part, be dictated by the pO_2_ concentration. Hence, Tat activity may be higher in the blood and intestine where pO_2_ levels range at higher levels (5.3–13.2% pO_2_)) compared to the lower levels reported for brain, skin and secondary lymphoid organs (< 4.4% pO_2;_) [38, 39].

We have previously developed Tat green fluorescent protein (GFP) fusion protein mutants to map the Tat region that contributes to the development of ADRs [21, 40]. The mutants include the full-length protein (Tat101GFP) and the product of the first exon (Tat72GFP), both of which have the NLS that restricts them to the nucleus. Additionally, a TatΔGFP mutant was created expressing the full-length protein with a deleted NLS that resulted in significant cytoplasmic distribution of the protein [21]. These constructs are expressed under the control of an inducible promoter so that the expression of these Tat mutant proteins approximated the level of expression achieved in HIV infected T cells. These cell lines express the recombinant proteins of the expected molecular weight and exhibit the expected intracellular distribution in the nucleus and cytoplasm depending on whether the NLS has been retained in a given mutant. These mutant forms of Tat enhance, to varying degrees, SMX-HA-mediated toxicity as we have observed in wildtype HIV infected T cells. These constructs, their induction characteristics, impact on cell viability and the degree to which they induce ROS have been reported in detail elsewhere [21, 40].

In the present study, we examined the changes in the cellular redox state of wildtype and mutant forms of Tat-expressing T cells in the absence and presence of SMX-HA by evaluating the disulfide proteome using a redox 2D SDS-PAGE system. We also determined the impact of SMX-HA and Tat on the oxidation level of peroxiredoxin 1, an endogenous redox sensitive protein. This report represents the first analysis of the cellular thiol proteome following HIV infection thereby defining the extent to which the virus alters the redox state at the molecular level under normoxic conditions.

## Methods

### Cell lines

The human T lymphocyte cell line Jurkat E6.1 (ATCC TIB-152), was obtained from the American Type Culture Collection. The cell line was chronically infected with HIV_IIIB_. The cells were maintained in complete RPMI 1640 medium (ThermoFisher Scientific, Burlington, Canada) supplemented with 1% L-glutamine, 20% fetal calf serum, 1 mM sodium pyruvate and 100 units/ml of penicillin and streptomycin (Thermo Fisher Scientific, Canada).

### Stable Tat and Tat deletion mutant-expressing cell lines

The plasmid construction, their transfection and the subsequent selection of Jurkat E6.1 T cells stably expressing wildtype Tat with and without GFP as well as the Tat deletion mutants fused to GFP was previously described in detail [21, 40]. The impact of wildtype Tat and each of the Tat deletion mutants on cell viability in the presence and absence of SMX-HA was also described previously [21]. Briefly, the Tat gene was PCR-amplified from the plasmid pSVTat that encodes the full-length Tat gene and was a kind gift from Dr. K.T. Jeang of the Molecular Virology Section, NIAID, NIH. The full-length Tat gene (Tat101), and Tat deletion mutants expressing just the first exon (Tat72) or the full-length Tat gene without the PTD/NLS (TatΔ) were cloned into the plasmid pEGFP-N1 (Clontech Inc., Mountain View, CA, USA), resulting in the genes for Tat101, Tat101GFP, Tat72GFP and TatΔGFP fusion proteins, respectively. These genes were subcloned into pBIG2i as previously described [40]. The result was pBIG Tat101, pBIG Tat101GFP, pBIG Tat72GFP, pBIG TatΔGFP and pBIG GFP as a control.

The pBIG Tat constructs or pBIG GFP were transfected into Jurkat-E6.1 T cells by nucleofection (Lonza, Walkersville, MD, USA) according to the manufacturer’s instructions and stable cell lines were generated as previously described [40]. The expression of both GFP and the TatGFP fusion proteins was induced by incubating the transfected cells with 0-1000 ng/ml doxycycline (Dox; Sigma, Canada). Dose response and time course of Dox induction were evaluated and characterized using flow cytometry or real-time PCR while the expression of the TatGFP constructs were confirmed via western blots [21].

#### SMX-HA

SMX-HA was synthesized by Katarina Sapeta in the laboratory of Dr. Michael A. Kerr at the Department of Chemistry (University of Western Ontario, London, ON, Canada). Sample purity was assessed using ^1^H NMR and was > 96%.

##### Sample preparation for slot blots and redox 2D gel electrophoresis

Jurkat pBIG cells (cultured under normoxic conditions) expressing wildtype or mutant forms of Tat fused to GFP (2.5-3 × 10^6^ cells) were induced with 0, 200, 400 or 1000 ng/ml of Dox for 40 h (viability > 95%) at which point the cells were washed twice with PBS by centrifugation at 400 x g for 5 min. The cells were suspended in HEPES buffer and seeded at a concentration of 0.3 × 10^6^ cells/ml. The cells were treated with 200 μM SMX-HA (a pharmacologically relevant concentration [41, 42]) or the vehicle (DMSO) (Millipore Sigma, Oakville, ON, Canada) at a final concentration of 0.5% then incubated at 37 °C for 2 h. The cells were subsequently collected and lysed in RIPA buffer containing 50 mM Tris-HCl pH 7.4, 150 mM NaCl, 1% Triton x-100, 1% sodium deoxycholate, 0.1% SDS, 1 mM EDTA (pH 8.0), complete mini protease inhibitor cocktail (Roche) and 40 mM iodoacetamide ([IA]; Millipore Sigma, Oakville, ON, Canada). Protein isolation was previously described [40] and the protein concentrations were determined using the DC Protein Assay (BioRad, Mississauga, ON, Canada) with bovine serum albumin (BSA) as the standard.

##### Slot blot analysis

Cells from the different cell lines were incubated with Dox for 40 h then incubated with varying concentrations of SMX-HA for 2 h under normoxic conditions. The cells were then collected and protein isolated as described above. Protein (1 μg) was resuspended in cold PBS and applied to slots on the slot blot microfiltration device (Bio-Dot SF Apparatus, Bio-Rad) according to the manufacturer’s instructions. Briefly, after assembly of the Bio-Dot SF Apparatus with the pre-wet nitrocellulose membrane, the appropriate wells were filled with sample solution. The flow valve was then adjusted to allow the entire sample to filter through the membrane by gentle vacuum and each well was washed with TBS (Tris-buffer saline, 50 mM Tris pH 7.6, 150 mM NaCl), again by pulling the wash liquid through by filtration. After the wells were completely drained, the membrane was removed. The membranes were subsequently blocked in TBS-T (TBS with 0.1% Tween 20) containing 5% dry milk for 1 h at room temperature with gentle shaking, and then probed overnight at 4 °C with an anti-SMX-HA rabbit polyclonal antibody [43] at a dilution of 1:30000 in TBS-T. This was followed by washing the membrane thrice for 10 min each time with TBS-T. The secondary antibody was an HRP-conjugated goat anti-rabbit antibody (Jackson ImmunoResearch Laboratories) diluted to 1:35000 in TBS-T. The blots were then treated with a chemiluminescent agent (Thermo Scientific Pierce ECL Western Blotting Substrate) and visualized by a Fluorchem Imager (Alpha Innotech/ Cell Biosciences). The blots were then stripped and probed with an anti-GAPDH monoclonal antibody (Sigma) at 1:5000 followed by a donkey anti-mouse conjugated to HRP (Jackson ImmunoResearch Laboratories) at 1:10000. Band intensity was determined by the AlphaView software (ProteinSimple, Santa Clara, CA, USA).

##### Non-reducing/reducing two-dimensional SDS/gel electrophoresis (R2D)

The two dimensional SDS-PAGE procedure was a modified version of that reported by Sommer and Traut [44]. Briefly, 80μg of protein dissolved in SDS sample buffer free of reducing agents and the proteins were resolved on a 1.0 mm thick, 10% polyacrylamide gel by subjecting the gel to electrophoresis at a constant current of 16 mA for 1 h followed by 1.5-2 h at a constant current of 24 mA. After electrophoresis, the lane containing the separated proteins was excised from the gel and immersed in freshly made SDS sample buffer containing 100 mM DTT (ThermoFisher Scientific, Burlington, ON, Canada) for 20 min at room temperature with gentle rocking. The gel strip was washed three times with 1× running buffer then immersed in SDS sample buffer containing 100 mM IA for 10 min at room temperature with gentle rocking. After another wash with 1× running buffer, the gel strip was applied horizontally to the top of a 1.5 mm thick 10% polyacrylamide gel of dimensions 20 cm(W) × 23 cm(L). Electrophoresis was carried out in the second dimension at a constant current of 135 mA for approximately 17 h. Each treatment sample of every cell line was subjected to the above R2D protocol at least three different times. Each set of 2D gels was silver-stained simultaneously according to the method of Shevchenko et al. [45]. A spot was recorded only if it was present in at least two of the three gels.

##### Mass spectrometry (MS) and protein identification

Gel spots from R2D gels were identified by mass spectrometry (MS) in the Functional Proteomics Facility of the Department of Biochemistry at the University of Western Ontario. Briefly, spots were excised and in-gel digested with sequence grade trypsin and lyophilized with a Mass Prep Automated Digestor (Waters, Milford, MA, USA). The lyophilized samples were dissolved in 10% acetonitrile and 0.1% trifluoroacetic acid, and then mixed with 5 mg/ml α-cyano-4-hydroxycinnamic acid (in 6 mM ammonium phosphate monobasic solution) at a 1:1 ratio. The samples were spotted in duplicate and the matrix-assisted laser-desorption/ionization (MALDI) MS spectra were acquired using a BiosystemsR 4700 Proteomics Discovery System (Applied Biosystems, Foster City, CA, USA), a matrix-assisted laser-desorption/ionization tandem time-of-flight mass spectrometer with TOF/TOF optics (MALDI-TOF).

The MALDI MS spectra were analyzed with the 4000 Series Explorer software (Applied Biosystems, Foster City, CA, USA) and processed into peptide mass fingerprints (PMF) using the Data Explorer software (Applied Biosystems, Foster City, CA, USA). Protein identification from these PMF was generated using a GPS engine connected to a Mascot server provided by the software manufacturer. PMF were compared to known peptide mass sequences in the NCBI (National Center for Biotechnological Information) database and samples with a PMF that has a high protein score (above 90%) were predicted to identify a specific protein.

##### Quantitative analysis, R2D gels

To determine the relative amount of the protein spots an area of interest was created around the spot and the corresponding pixel intensity values in that area were obtained. These values were corrected for background. The value obtained for the control, uninfected Jurkat E6.1 cells was set as 100% and the value for the same spot in the other cell lines calculated as a percent of this value. To determine the molecular weights of the unknown protein spots, the values and positions on the image of known molecular weight markers were used as reference points. The analyses were both carried out using the AlphaView software.

##### Western blot analysis of peroxiredoxin 1

Jurkat pBIG TatGFP cells (cultured under normoxic conditions) were induced with 0, 200, 400 or 1000 ng/ml of Dox for 40 h, washed twice with PBS, resuspended in HEPES buffer and seeded at a concentration of 0.3 × 10^6^ cells/ml. The cells were then treated with 100 μM SMX-HA or the vehicle (0.5% DMSO, *v*/v) (Sigma-Aldrich, Canada) and incubated at 37 °C for 2 h. This was followed by another 2 h incubation in complete RPMI at 37 °C. The cells were subsequently harvested, washed in PBS and lysed in RIPA buffer containing 50 mM Tris-HCl pH 7.4, 150 mM NaCl, 1% Triton X-100, 1% sodium deoxycholate, 0.1% SDS, 1 mM EDTA (pH 8.0) and complete mini protease inhibitor cocktail (Roche, Mississauga, ON, Canada). Protein was isolated [40] and the concentration determined using the DC Protein Assay (BioRad, Mississauga, ON, Canada) with bovine serum albumin (BSA) as the standard. Protein (25 μg) was dissolved in sample loading buffer free of reducing agents and resolved on 15% SDS-PAGE then transferred onto PDVF membranes. Immunoblot analysis was performed using a mouse monoclonal antibody to peroxiredoxin 1 (Prx1) (1:5000, AbFrontier, Cedarlane Laboratories Limited, Burlington, ON, Canada) and a secondary antibody conjugated to horseradish peroxidase (1:20000, Jackson ImmunoResearch, West Grove, PA). Immunoreactivity was visualized with enhanced chemiluminescence (ThermoFisher Scientific, Burlington, ON, Canada) and the Odyssey Fc Imaging System (LI-COR Biosciences, Lincoln, NE, USA). Band densitometry was quantified using Image Studio Lite (LI-COR Biosciences, Lincoln, NE, USA).

### Statistics

Slot blot data were analyzed in GB-Stat (Dynamic Microsystems, Inc., Silver Spring, MD USA). Means and standard errors (SE) were used to report continuous variables. A two-way ANOVA with Tukey’s post hoc procedure was used to compare mean differences between groups for different concentration levels of each cell line. In Table 4, a two-way ANOVA with Bonferroni post-tests was used to compare the mean differences for Prx1 between the cell lines for each treatment.

## Results

### Quantification of SMX-HA-induced haptenation in tat-expressing Jurkat T cells

**Fig. 1:**
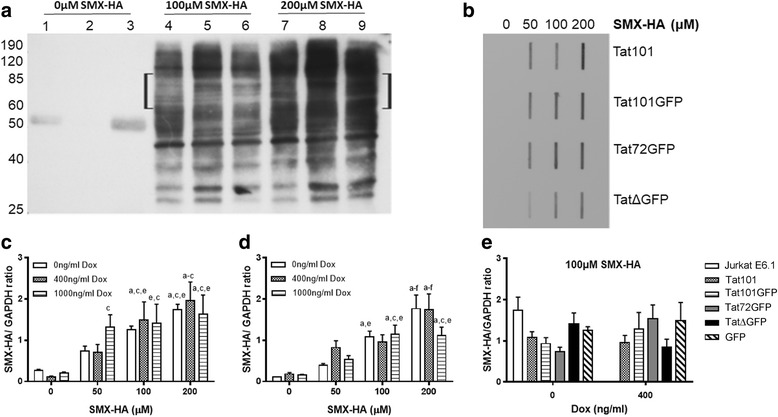
Representative western blot and slot blot data showing SMX-HA-induced haptenation. **a** Western blot of cells treated with 0 (lanes 1–3), 100 μM (lanes 4–6) and 200 μM (lanes 7–9) SMX-HA for 2 h and resolved on 12% SDS-PAGE. Lanes 1, 4, 7: Jurkat E6.1; lanes 2, 5, 8: Tat101GFP; lanes 3, 6, 9: Tat72GFP. **b** Total protein from cells treated with various concentrations of SMX-HA and applied onto a membrane via a slot blot apparatus. Both membranes were blotted with anti-SMX-HA antibody. The band intensities were measured digitally and the ratio of SMX-HA haptenation to GAPDH content in the corresponding sample was calculated. (C and D) The SMX-HA/GAPDH ratio increases with increasing concentration of SMX-HA for GFP (**c**) and wildtype Tat101 (**d**) expressing Jurkat E6.1 cells. Significance was defined as follows: a, c, e *P* < 0.05 vs. 0, 400, 1000 ng Dox ml^− 1^ at 0 μM SMX-HA, respectively. b, d, f *P* < 0.05 vs. 0, 400, 1000 ng Dox ml^− 1^ at 50 μM SMX-HA, respectively. g *P* < 0.05 vs. 200 ng Dox ml^− 1^ at 100 μM SMX-HA. **e** Expression of wildtype Tat101 and Tat mutants does not result in significantly different SMX-HA/GAPDH ratios compared to untransfected Jurkat E6.1 and GFP expressing cells. Data are expressed as mean of three independent experiments

To determine if expression of the HIV-1 protein Tat in Jurkat T cells affects SMX-HA-induced haptenation, western and slot blot experiments were conducted. Tat cell lines were either untreated or treated with a concentration of Dox that induced expression of Tat RNA similar to that observed in HIV-infected Jurkat T cells [40]. Following a 2 h exposure to increasing concentrations of SMX-HA (0, 50, 100 and 200 μM), protein isolated from each Tat-Jurkat cell line was subjected to western blotting with an anti-SMX-HA antibody to visualize the haptenation pattern. A representative example is shown (Fig. 1a) that illustrates the qualitative differences in protein haptenation banding patterns between the different cell lines and concentrations of SMX-HA employed (bracket, Fig. 1b). To determine if there were quantitative differences in total protein SMX-HA haptenation between cell lines expressing wildtype or mutant Tat constructs, slot blots were performed (representative blot, Fig. 1b) and the hapten protein/GAPDH ratios determined (representative data presented in Fig. 1c-e). Accordingly, ratios increased at least 2.5- to 3-fold when the parental Jurkat E6.1, GFP-expressing Jurkat E6.1 and each of the Tat-expressing Jurkat E6.1 cell lines were treated with 50 μM SMX-HA. Moreover, these ratios tended to increase when the SMX-HA concentration was increased to 100 or 200 μM (representative data shown in Fig. 1c and d). Importantly, there was no evidence that the induction of the Tat constructs resulted in significant qualitative and quantitative differences in total SMX-HA-mediated haptenation at any tested SMX-HA concentration (representative data shown in Fig. 1e).

### Detection of ROS-sensitive thiol proteins

**Fig. 2:**
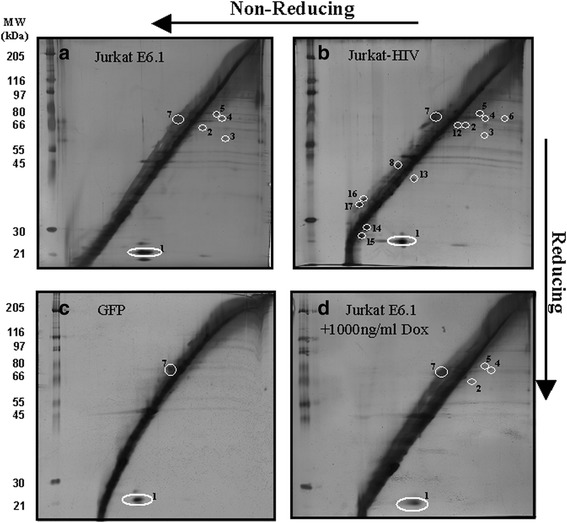
R2D SDS-PAGE of thiol proteins from control cell lines. Samples from (**a**) Jurkat E6.1, (**b**) Jurkat-HIV, (**c**) GFP and (**d**) Jurkat E6.1 + 1000 ng/ml Dox. The sample from panel D was incubated with 1000 ng/ml doxycycline for 40 h. Cells from each cell line were then treated with 0.5% DMSO for 2 h, collected and protein was isolated. Protein lysate (85 μg) was loaded onto the first dimension gel and run for 3 h followed by an overnight run of the second dimension gel. On the left side of the diagonal on each gel are molecular weight protein standards that are enumerated to the left of panels **a** and **c**. The gels shown in panels **a** and **b** are duplicated and used as a reference in panels **a** and **b** of Fig. 3 and Additional file 1: Figure S1 and Additional file 2: Figure S2

Due to the many SMX-HA haptenated proteins revealed by western blot analysis (Fig. 1), we next focused on documenting oxidative changes (i.e. enhanced formation of PSSP) arising from the treatment with the electrophilic oxidative stressor, SMX-HA and the presence of the HIV-1 Tat protein or its deletion mutants. To explore this, a R2D system was employed as it provides a direct means to identify proteins that are redox regulated via oxidation of reactive protein cysteine thiols through formation of protein-protein homomeric disulphides (PSSP) and heteromeric disulphides (PSSP’) [33]. Cell lysates from 2.5-3 × 10^6^ cells were analyzed by R2D. Proteins containing reduced protein thiol (P-SH) residues prior to first dimension gel application resolve on the diagonal line (Fig. 2a) by molecular weight subsequent to the gel runs in the second dimension, as dithiothreitol (DTT) does not reduce these proteins further. In contrast, protein-protein mixed disulphides, following the first electrophoresis run, are reduced by treatment with DTT in the first dimension gel matrix so that the protein thiols formed upon reduction of intermolecular disulphide bonds are resolved below the diagonal line (Fig. 2a, spots 2–5). This is due to the dissociation of protein dimeric disulphides and the attendant decrease in molecular mass after reduction revealed in the second dimension. However, proteins with intramolecular disulphide bonds after reduction with DTT have an apparent increase in molecular mass and run above the diagonal line after second dimension electrophoresis. R2D gel electrophoresis does not resolve S-glutathionylated proteins because of the small molecular weight change resulting from the addition of GSH to the protein, and the removal of GSH during DTT reduction. Each treatment condition was analyzed on the R2D gels in at least three independent experiments and a spot was recorded only if it was present in at least two of the three gels. Table 1 catalogues the protein spots resolved on the R2D gels.

**Table 1:**
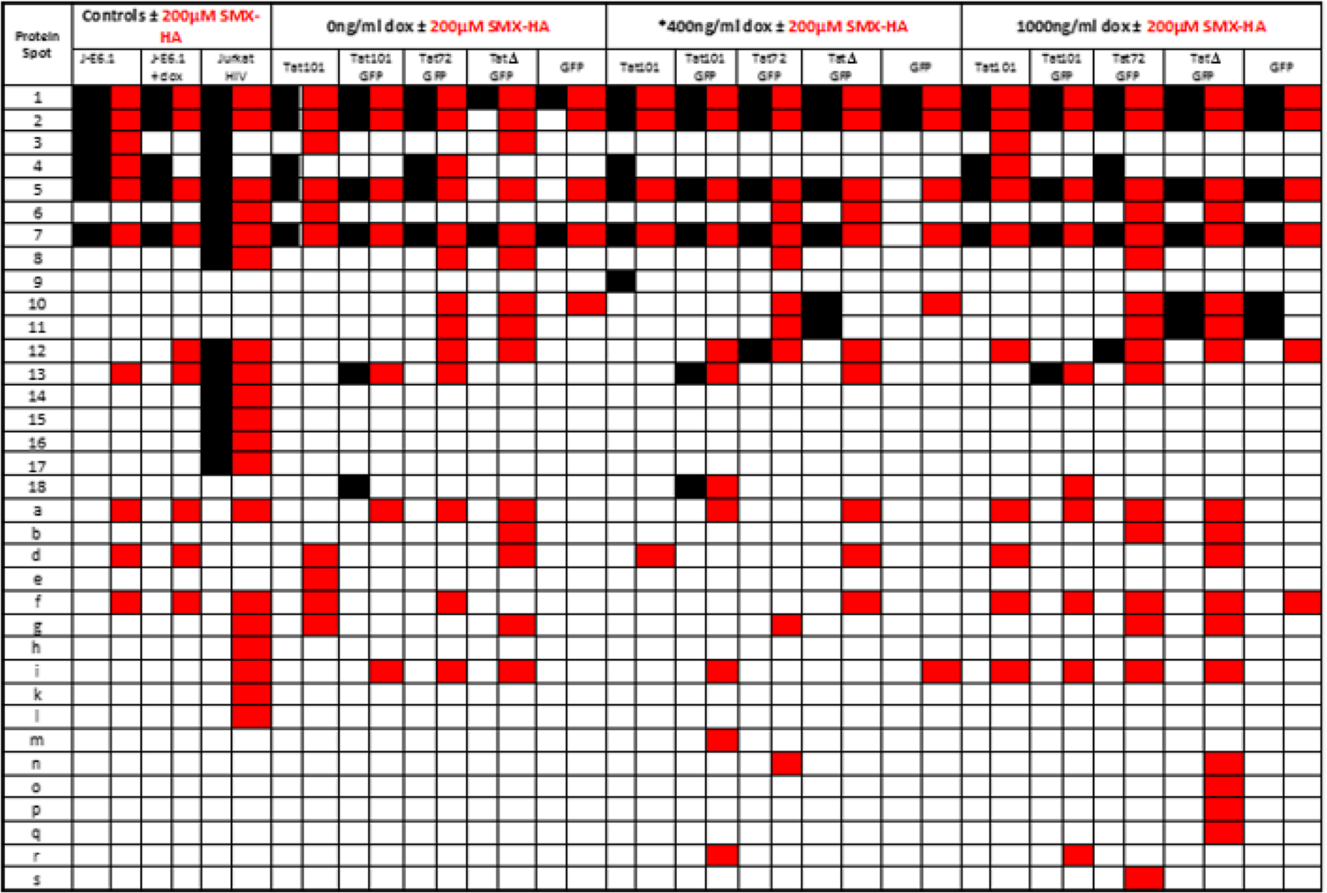
Summary of protein-protein mixed disulphide-derived spots found in the R2D gels of lysates from the cell lines used in this investigation

Black squares indicate the presence of the corresponding protein spot in that cell line and red squares represent the presence of the corresponding protein spot in that cell line only after treatment with 200 μM SMX-HA. J-E6.1 + Dox: Jurkat E6.1 cells incubated with 1000 ng/ml doxycycline (Dox). Jurkat HIV: Jurkat E6.1 cells infected with HIV. *Concentrations of Dox selected to induce a level of TatGFP mRNA equivalent to that seen in HIV-infected Jurkat T cells. For the Tat101GFP and Tat72GFP cell lines 200 ng/ml Dox was used, whereas 400 ng/ml Dox was used for all other cell lines

### HIV infection but not Tat expression alone increases oxidation of protein thiols

Analysis of the different conditions indicated that overall the pattern of protein spots was similar with most of the spots resolving between 55 and 97 kDa. Representative 2D gels of protein samples are presented for different control cell lines (Fig. 2), including the untransfected parent cell line Jurkat E6.1 incubated with or without 1000 ng/ml Dox (Fig. 2a and d), HIV infected Jurkat T cells (Fig. 2b) and the stably transfected Jurkat T cell line expressing only EGFP (Fig. 2c). Observable differences were present in both the protein abundance of spot no. 1 and the number of individually resolved protein spots (e.g., Fig. 2a vs 2b). Of the control samples, the Jurkat-HIV R2D protein gels revealed the greatest number of PSSP and PSSP’-derived spots (Fig. 2b and Table 1), indicative of increased oxidative stress and/or the presence of novel redox-related proteins in HIV-infected cells. The majority of the protein disulphides formed have intermolecular bonds as they resolved below the diagonal line on R2D. The Jurkat E6.1 cell line (Fig. 1a) had seven identical protein spots to Jurkat-HIV cells while Jurkat E6.1 cells incubated with 1000 ng/ml Dox only showed five proteins common with Jurkat-HIV cells (Fig. 2d). Jurkat cells transfected with EGFP had the fewest spots (Fig. 2c). Data from the un-induced (0 ng/ml Dox) samples from the different Tat transfected cell lines are summarized in Table 1 with representative gels shown in Additional file 1: Figure S1. They revealed very similar patterns and numbers of spots as observed for the Jurkat E6.1 and GFP transfected control cell lines.

**Fig. 3:**
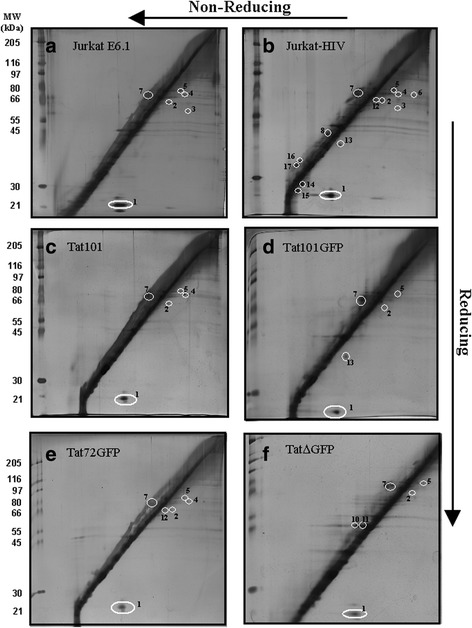
R2D SDS-PAGE of thiol proteins formed by reduction of cellular protein-protein mixed disulphides. Samples from (**a**) Jurkat E6.1, (**b**) Jurkat-HIV, (**c**) Tat101, (**d**) Tat101GFP, (**e**) Tat72GFP and (**f**) Tat∆GFP, with panels **c**-**f** induced for 40 h with 1000 ng/ml Dox prior to DMSO treatment. The R2D gels in panels **a** and **b** are replicas of those seen in Fig. 2a and b respectively. Cells from each of the various lines were treated with 0.05% DMSO for 2 h, collected and the protein was isolated. Protein lysate (85 μg) was loaded onto the first dimension gel and run for 3 h followed by an overnight run of the second dimension gel. On the left side of the diagonal on each gel are molecular weight protein standards that are enumerated to the left of panels **a**, **c** and **e**

Next, we examined the gel pattern of the different cell lines treated with either 400 or 1000 ng/ml Dox that induce Tat or TatGFP mRNA expression to equivalent or elevated levels, respectively, present in HIV-infected Jurkat cells [21]. Generally, this resulted in the appearance of the same protein spots and occasionally in the loss of a protein spot previously seen in R2D gels of the 0 ng/ml Dox samples (Fig. 2, Table 1 and Additional file 2 Figure S2). Over expression of Tat induced with 1000 ng/ml Dox in the transfected cell lines (Fig. 3c-f) did not drastically change the number or identity of proteins that resolved away from the diagonal compared to 0 ng/ml Dox (Additional file 1: Figure S1) or when induced with 400 ng/ml Dox (Additional file 2 Figure S2). All the resolved proteins were equivalent to those seen from the parental cell line or the HIV infected cell line (Table 1). The exception was the Tat∆GFP cell line where two novel proteins with approximate molecular weights of 44 kDa (Fig. 3f, spots 10 and 11) were found above the diagonal line. For this cell line, Tat∆GFP induction at 1000 ng/ml Dox resulted in protein spots 10 and 11 tending to appear with greater intensity than seen following induction at 400 ng/ml Dox (Fig. 3f vs Additional file 2: Figure S2F), the concentration that generates Tat mRNA equivalent to that in HIV-infected cells [21]. Thus, in the absence of SMX-HA, wild type Tat and the Tat deletion mutants do not appear to have a significant impact on the disulphide proteome.

### The combination of high tat expression and SMX-HA treatment produced the highest number of oxidized protein-protein mixed disulphides (PSSP and PSSP’)

**Fig. 4:**
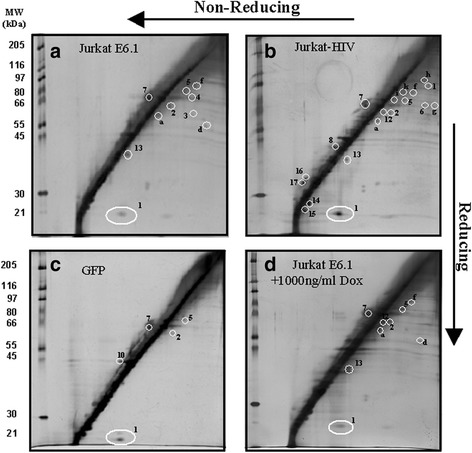
R2D SDS-PAGE of thiol proteins from control cell lines treated with SMX-HA. Samples from (**a**) Jurkat E6.1, (**b**) Jurkat-HIV, (**c**) GFP and (**d**) Jurkat E6.1 + 1000 ng/ml dox. The sample are from panel D was incubated for 40 h with 1000 ng/ml Dox prior to drug treatment. Cells from each of the various lines were then treated with 200 μM SMX-HA for 2 h, collected and the protein was isolated. Protein lysate (85 μg) was loaded onto the first dimension gel and run for 3 h followed by an overnight run of the second dimension gel. The gels shown in panels A and B are duplicated and shown as a reference in panels A and B of Fig. 5 and Additional file 1: Figure S1 and Additional file 2: Figure S2. On the left side of the diagonal on each gel are molecular weight protein standards that are enumerated the left of panels **a** and **c**

Cell lines treated with 200 μM SMX-HA showed a 2- to 3-fold increase in the number of proteins resolved below the diagonal line in addition to the oxidized protein spots observed in the absence of SMX-HA (Fig. 4, spots 1–18, Table 1). The proteins that resulted from the oxidative stress uniquely resulting from the addition of electrophilic SMX-HA are lettered a-s (Fig. 4a-d; Additional file 3: Figure S3A-F, Table 1). These haptenated proteins could not be identified by mass spectrometry due to limiting amounts.

Treatment of the parental cell line Jurkat E6.1 with SMX-HA did not affect the resolution of any of the numbered proteins, but added new oxidized proteins to the profile (Table 1 and Fig. 4a). SMX-HA-treatment of Jurkat E6.1 T cells induced with 1000 ng/ml Dox (Fig. 4d) tended to reduce the abundance of the protein in spots 3 and 4 indicating lower amounts of these proteins were oxidized (i.e. occur as PSSP). The treatment of the HIV-infected Jurkat cell line with SMX-HA caused the loss of some proteins, but also dramatically increased the number of protein spots including two unique protein spots k and l (Table 1 and Fig. 4b). R2D gel electrophoresis of samples from the cell line expressing EGFP (the negative control) resulted in the resolution of proteins common to the parental cell line, but none of the novel lettered proteins (Table 1 and Fig. 4c).

**Fig. 5:**
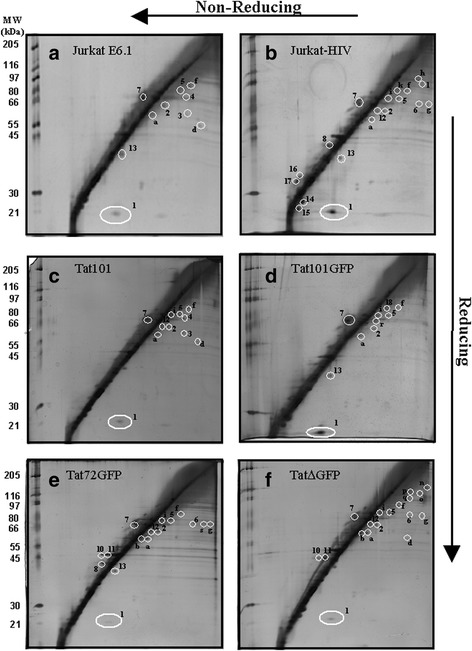
R2D SDS-PAGE of thiol proteins from cell lines treated with SMX-HA. Samples from (**a**) Jurkat E6.1, (**b**) Jurkat-HIV, (**c**) Tat101, (**d**) Tat101GFP, (**e**) Tat72GFP and (**f**) Tat∆GFP, the last four panels showing samples induced for 40 h with 1000 ng/ml doxycycline prior to drug treatment. The R2D gels in panels **a** and **b** are replicas of those in Fig. 4a and b, respectively. Cells from each of the various lines were then treated with 200 μM SMX-HA for 2 h, collected and the protein was isolated. Protein lysate (85 μg) was loaded onto the first dimension gel and run for 3 h followed by an overnight run of the second dimension gel. On the left side of the diagonal on each gel are molecular weight protein standards that are enumerated to the left of panels **a**, **c** and **e**

Treatment of the uninduced cells (0 ng/ml Dox) with SMX-HA resulted in the Tat72GFP and Tat∆GFP cell lines displaying the most protein spots from the Tat-transfected cell lines (Table 1 and Additional file 3: Figure S3E and F). The majority of these proteins contain intermolecular disulphide bonds, running below the diagonal line of unresolved proteins. Maximal induction of Tat expression with 1000 ng/ml Dox generated the most protein spots, reflecting enhanced oxidative stress. In addition to the resolution of previously documented proteins, the Tat∆GFP cell line also produced three unique proteins o, p and q (Table 1 and Fig. 5f).

### Peroxiredoxins as a measure of cellular oxidative stress in Jurkat T cells

The R2D gel system revealed a number of proteins with intermolecular disulphide bonds (below the line), but most notably there were intense spots detected just below 30 kDa (elliptically circled in white) and labelled as spot 1 in all R2D Figure panels. After isolation from the R2D gel, both the top and bottom proteins were in-gel digested with trypsin and identified by MALDI-TOF mass spectrometry as Prx1 and Prx2, respectively (Tables 2 and 3).

**Table 2:** Mass spectrometric peptide mass fingerprinting (MS PMF) sequence data for protein spot 1

Peptide Information						
Calc. Mass	Observ Mass	± da	± ppm	Start Seq.	End Seq.	Sequence
819.4207	819.4188	−0.0019	−2	152	158	SVDETLR
894.4315	894.4318	0.0003	0	121	128	ADEGISFR
980.5311	980.5272	−0.0039	−4	8	16	IGHPAPNFK
1107.6045	1107.5933	−0.0112	−10	111	120	TIAQDYGVLK
1196.631	1196.63	−0.001	−1	159	168	LVQAFQFTDK
1211.6742	1211.6702	−0.004	−3	141	151	QITVNDLPVGR
1359.7994	1359.7931	−0.0063	−5	129	140	GLFIIDDKGILR
1524.7917	1524.7761	−0.0156	−10	2	16	SSGNAKIGHPAPNFK

Data was obtained from MASCOT®, a search engine that compared the PMF of tryptic digests of protein spot 1 against an NCBI database of known protein masses to identify the disulphide bonded protein separated by R2D gels. The protein was determined to be peroxiredoxin 1 (*Homo sapiens*), a member of the peroxiredoxin family of antioxidant enzymes

**Table 3:** MS PMF identification of a protein that forms intermolecular disulphide bonds in Jurkat T cells after separation by R2D SDS-PAGE

Protein Spot	Protein Name	Accession No.	Protein Score %	Protein MW	Protein PI
1	Peroxiredoxin 1 [*Homo sapiens*]	gi_∣_55,959,887	91	19,134.7	6.41

The Prx proteins in the parental Jurkat E6.1 cell line consistently showed the highest abundance relative to those in the other control and Tat-expressing cell lines. The densitometric value of these Prx proteins served as a benchmark (100%) against which the Prx proteins from the other cell lines were compared. The untreated samples (0μM SMX-HA) from the Tat-expressing cell lines were found to be reduced to 31–42% in relation to the intensity of protein Prx1 (spot no. 1; Table 4 and Additional file 1: Figure S1). The relative abundance of Prx in the untreated Jurkat HIV samples (0μM SMX-HA) was also reduced to 44% in relation to Prx1 (spot no. 1) of untreated Jurkat E6.1 cells (Table 4). The R2D gels of the different cell lines induced with 1000 ng/ml Dox and treated with 200μM SMX-HA revealed a further decrease in the relative abundance of Prx in all cell lines (Table 4 and spot no.1, Figs. 4 and 5; Additional file 3 Figure S3 and Additional file 4 Figure S4). In this instance, the Prx abundance for the Jurkat HIV cell line decreased to 33% followed by the Tat101 cell line, which decreased to 23% in relation to untreated Jurkat E6.1 cells (0 Dox, 0 SMX-HA). The Tat72GFP and Tat∆GFP cells saw Prx abundance decrease to 10% in relation to Prx1 (spot no. 1) in untreated Jurkat E6.1 cells, suggesting they were most affected by SMX-HA-induced oxidative stress (Table 4 and Additional file 3: Figure S3). Upon Tat induction (400 ng/ml Dox), the Tat101GFP cell line showed an increased intensity for Prx. By comparison in the presence of SMX-HA, the abundance for Prx in the Tat101, Tat72GFP and Tat∆GFP transfected cell lines tended to be decreased further (Table 4).

**Table 4:** Spot intensities of the peroxiredoxin proteins

SMX-HA	0μM			200μM		
Dox (ng/ml)	0	400	1000	0	400	1000
Jurkat E6.1	100 ± 1	n/d	n/d	33 ± 1.4	n/d	37 ± 2.0
Jurkat HIV	44 ± 2.2***	n/d	n/d	33 ± 0.6	n/d	n/d
Tat101	31 ± 1.1***^†^	46 ± 1.4***	35 ± 0.1***	31 ± 4.4	8.5 ± 0.3***^†††^	23 ± 0.5***
Tat101GFP	34 ± 1.4***	30 ± 0.6***^†^	32 ± 0.8***	12 ± 2.4***^†††^	41 ± 1.4	18 ± 2.5***^†^
Tat72GFP	44 ± 4.1***	41 ± 0.3***	39 ± 1.0***	7.8 ± 0.1***^†††^	6.7 ± 1.2***^†††^	7.8 ± 0.7***^†††^
TatΔGFP	33 ± 4.0***	43 ± 0.6***	35 ± 0.6***	13 ± 1.1**^†^	10 ± 0.1***^†^	9.5 ± 0.4***^†^

Values for each of the R2D gels were calculated as a percentage of the spot intensity for protein 1 of the Jurkat E6.1 sample. n/d: not done. ^***^p < 0.001, ^**^p < 0.01 vs. Jurkat E6.1 and ^†††^p < 0.001, ^†^*p* < 0.05 vs. Jurkat HIV

### Peroxiredoxin 1 redox changes

Peroxiredoxins (Prx) are ubiquitous and highly expressed peroxidases that use reversibly oxidized cysteine residues to reduce peroxides (H_2_O_2_) [46]. Given the abundance of Prx1 seen in the R2D gels as well as the fact that Prx1 serves as a sensitive marker of oxidation in cells [46], we used non-reducing SDS-PAGE and an anti-Prx1 antibody to examine the oxidation state of Prx1 after expression of the different Tat constructs and SMX-HA treatment. This method relies on the fact that cell lysis in the absence of an alkylating agent will result in the oxidation of reduced Prx leading to the formation of disulfide-linked dimers of ~ 44 kDa, while hyperoxidized Prx1 is unable to dimerize and therefore will remain as a monomer at ~ 22 kDa [47]. The ratio of monomer to dimer serves as a specific marker of oxidative stress [48, 49].

**Fig. 6:**
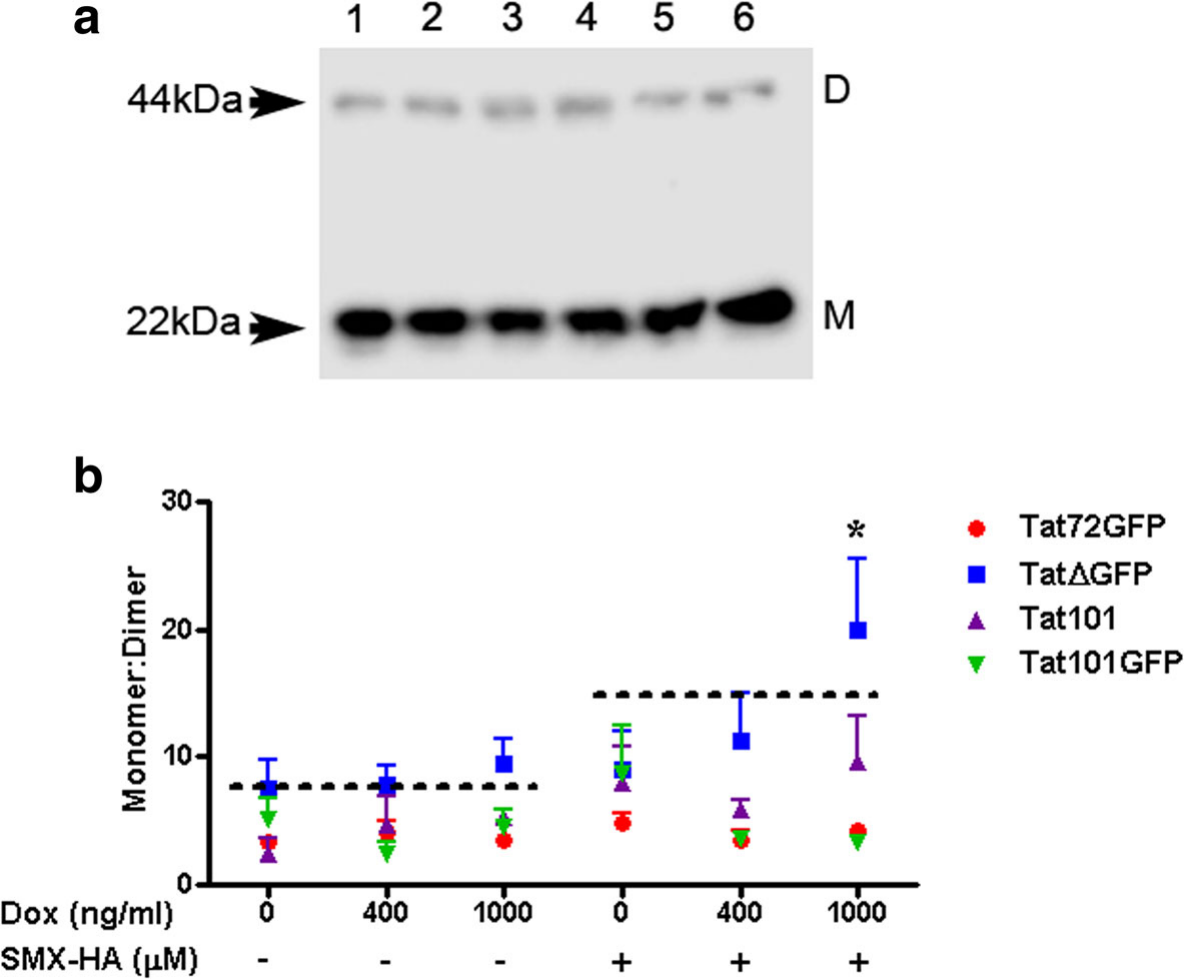
The effect of Tat expression and SMX-HA-mediated oxidative stress on Prx1 oxidation in Jurkat T cells. Cells from the various cell lines were induced with 0, 400 or 1000 ng/ml Dox for 48 h, treated with 0. 5% DMSO or 100μM SMX-HA for 2 h followed by another 2 h incubation in media. The cells were then collected, cell lysates prepared and 25μg protein were dissolved in non-reducing loading buffer. The protein samples were run on 15% SDS-PAGE and transferred to membranes that were then probed with antibodies to Prx1. **a** A representative immunoblot showing oxidized dimers (labelled D on blots) and hyperoxidized monomers (labelled M on blots). **b** Quantification of the Prx1 monomer:dimer ratio in the Tat-expressing cell lines, presented as means ± S.E

The oxidation of Prx1 was examined in the four Tat-expressing cell lines. Cells were induced with Dox for 48 h prior to incubation with vehicle (DMSO) or 100 μM SMX-HA. Lower concentrations of SMX-HA (100 μM) were used to maintain cell viability, particularly in cells expressing Tat∆GFP as they are highly sensitive to SMX-HA [21]. Differential Tat expression in the absence of SMX-HA did not result in significant changes of the Prx1 monomer/dimer ratio in any of the cell lines (Fig. 6). However, induction of Tat101GFP expression in the presence of 100 μM SMX-HA decreased the monomer/dimer ratio while the ratio for the Tat∆GFP cell line increased under these treatment conditions. The maximal level of Tat∆GFP expression (1000 ng/ml Dox) combined with SMX-HA treatment led to a significant increase of the monomer/dimer ratio compared to the other Tat-expressing cell lines (Fig. 6), suggesting the Tat∆GFP cell line is more susceptible to the hyperoxidation of Prx1 under those conditions.

## Discussion

The Hapten Hypothesis is one of the most widely accepted mechanisms for the development of hypersensitivity ADRs. This hypothesis contends that the initial step(s) in the pathogenesis of hypersensitivity ADRs involve(s) the metabolic bioactivation of the drug to a hapten with inherent immunogenicity that then elicits an undesired immune response. In the case of SMX, its reactive oxidative metabolite (SMX-HA) can be further oxidized to SMX-NO, which, subsequent to covalent binding to protein(s), is postulated to be a hapten that elicits an immune response [43, 50, 51]**.** Our results demonstrate that the degree to which haptenation occurs is dependent on the concentration of SMX-HA but independent of HIV-I infection or whether wildtype or mutant forms of Tat are expressed in the Jurkat cell lines. Hence, we focused on the ability of SMX-HA/SMX-NO to induce intracellular oxidative stress by generating ROS [21, 52] and ROS-dependent GSH depletion rather than the immunological consequences of haptenation. Given the high incidence (up to 50%) of hypersensitivity ADRs in the HIV population [41] as well as Tat’s ability to down-regulate three regulators of ROS (manganese-SOD, selenoglutathione peroxidase and glutathione synthase), HIV-infected cells as well as cells subjected to extracellular Tat would be more sensitive to the effects of ROS and GSH depletion on exposure to SMX-HA [11, 53].

For this study we exposed wildtype, HIV-infected and Tat mutant transfected Jurkat cell lines to concentrations of SMX-HA that ranged from 0 to 200 μM. Patients treated with SMX (0.5–2 g/dose) or its derivatives have plasma concentrations that range widely from 40 to 600 μM [54–56]. All patients treated with SMX also appear to contain SMX-HA [57]. Cells, including circulating peripheral blood mononuclear cells that take up SMX-HA, further metabolize it to the more reactive metabolite, SMX-NO. This nitroxide metabolite is likely the chemical species that haptenates proteins [58]. Exposure to HIV or to the Tat constructs increases oxidative stress which, in turn, results in reduced intracellular GSH [9], promoting the conversion of SMX-HA to SMX-NO. In vivo, about 5% of the input SMX is converted to reactive metabolites SMX-HA and SMX-NO [24, 25] but how this relates to SMX-HA and SMX-NO concentrations in the blood and in secondary lymphoid tissues has not been measured. Using estimates of total plasma-derived water content (~ 3 L) [59, 60] and that up to 5% of a 0.5–2 g dose of SMX undergoes [24, 25] conversion, the combined concentration of SMX-HA/SMX-NO might reach the 30-120 μM range under ideal conditions.

Human leukocytes exposed in vitro to various concentrations of SMX-HA (1-800 μM) demonstrate reductions in leukocyte proliferation and viability as well as functional alterations [42, 59, 61–63]. In addition, leukocytes exposed to similar SMX-HA concentrations were sufficiently haptenated on the cell surface to elicit proliferative responses from T cells taken from humans exhibiting SMX hypersensitivity reactions [64]. In vitro, SMX fails to elicit such responses. This study and our previous studies examining the effects of HIV infection and Tat expression on SMX-mediated ADRs employed concentrations of SMX-HA (50-200 μM) in the lower end of the concentration range experimentally utilized in the literature. We selected this range in order to minimize the effect of SMX-HA on cell viability and because the selected range overlaps with our estimates and those of other estimates of what likely represents pharmacologically relevant concentrations of SMX-HA [57, 59, 65]. In AIDS patients, changes in drug metabolism that could contribute to elevated levels of the toxic metabolites SMX-HA and SMX-NO due to reductions in GSH content may place them at an elevated risk for SMX adverse drug reactions due to the higher doses of SMX employed to treat opportunistic infections [59, 66, 67].

The main targets of small molecule electrophiles such as SMX-HA are amino acid side chains with nucleophilic properties such as the ionized cysteine sulfhydryl group, the N-terminal amino group and the histidine imidazole group [68]. As post-translational modification of activated cysteine residues of proteins is critical in redox regulation, we evaluated the effect of SMX-HA/SMX-NO on the oxidation of protein cysteine thiols using R2D gel electrophoresis to test for increased oxidation of redox-regulated proteins to PSSP and PSSP’ protein-protein disulphides, a critical component of the disulfide proteome.

The GFP cell line had the fewest protein-protein disulphide-derived spots. The Tat∆GFP cell line consistently displayed more protein spots in the R2D gels than the other Tat-expressing cell lines. This likely relates to the cellular distribution of the Tat constructs. With the exception of the aforementioned Tat∆GFP construct, which lacks an NLS, the other Tat proteins are predominantly nuclear [21]. While Tat∆GFP also has a nuclear presence, the loss of the NLS renders it cytoplasmic at high levels where, as we have shown previously [21], it has additional effects not seen in the other Tat-expressing cell lines. Indeed, the TatΔGFP construct significantly decreases Jurkat T cell viability in the presence of SMX-HA [21].

The use of the soluble fraction of the protein samples in the R2D gels provides further enrichment for cytoplasmic localized protein disulphides and thus Prx1 (spot 1), the main cytoplasmic form of Prx1 in Jurkat cells and the predominant spot on the R2D gels in control cells. Spot 1 was confirmed as Prx1 by mass spectrometry, which is in agreement with the identification made by others [33, 36]. Peroxiredoxins (Prx) are a family of antioxidant proteins approximately 21-30 kDa in size that use specialized cysteines to decompose peroxides. The mammalian Prx family has six members (Prx1 to Prx6) differentially expressed in different subcellular compartments with Prx1 as the major cytoplasmic form in Jurkat T cells [69]. Prx1 is a 2-Cys Prx that exists as non-covalently-linked homodimers and carries out peroxide detoxication in three main steps: peroxidation, resolution and recycling [70, 71]. The Prx catalytic cycle begins with H_2_O_2_ oxidizing the peroxidatic (reactive) cysteine of a Prx molecule to form sulfenic acid (Prx-SOH), which can condense with the resolving cysteine of a neighbouring Prx molecule to form an intermolecular disulfide bond [48]. The resulting Prx dimer can be reduced by the thioredoxin system in eukaryotes [46, 48]. However, under high H_2_O_2_ concentrations, an alternative to disulfide formation can occur, leading to the hyperoxidation of Prx to the sulfinylated (Prx-SO_2_H) and sulfonylated (Prx-SO_3_H) forms [48]. Although these hyperoxidized forms can be slowly reduced by sulfiredoxin in an ATP-dependent reaction in vivo, under our assay conditions they remain enzymatically inactivated resulting in the inhibition of peroxidase function [48, 72]. It has been proposed that inactivation of Prx may be crucial for H_2_O_2_ to accumulate and react with cysteines in proteins that are normally slower to react than Prx, thereby enabling the redox reactions required to relay downstream signalling [73].

With excess ROS, Prxs becomes hyperoxidized during the catalytic cycle, leading to loss of Prx thiols and enzyme inactivation [74–77]. The decrease of the Prx1 protein disulphide (spot 1) seen in HIV-infected cells confirms that these cells are under more oxidative stress than the parental Jurkat E6.1 cells, consistent with HIV infection [31, 78, 79]. The further decrease in Prx1 protein levels in the Tat-expressing cells compared to the HIV-infected cells suggests there was inactivation of the enzyme due to further, uncompensated, oxidation and that the expression of Tat or its deletion mutants resulted in additional oxidative stress (vs HIV infection) within the Jurkat T cells. This would be enhanced in the presence of SMX-NO which reacts rapidly with GSH to form a labile semimercaptal derivative, consuming intracellular GSH [50]. This unstable SMX-semimercaptal is isomerized to a more stable sulfinamide with the release of SMX-HA. The sulfur atom of the sulfinamide is derived from GSH and the released SMX-HA can deplete another molecule of GSH upon re-oxidation to SMX-NO (i.e. a futile metabolic cycle).

Exposure of the different Tat-expressing cell lines to pharmacologically-relevant concentrations of SMX-HA also lead to additional hyper-oxidation and inactivation of the Prx enzymes in all the Tat-expressing cell lines studied. For the cell lines expressing Tat101, Tat72GFP and Tat∆GFP, the decrease in the abundance of the Prx disulphide spot after treatment with SMX-HA was more pronounced than that seen in the HIV-infected cells. This suggests that cell lines chronically infected with HIV may compensate for the loss of the Prx enzymes produced by the oxidative stress of SMX-HA by mechanisms yet to be defined. One probable reason for the apparent decrease in the amount of the Prx disulphide in the Tat-expressing cells after the treatment with SMX-HA is that the Tat protein is able to suppress the expression and activity of other important cellular antioxidants such as GSH and Mn-SOD [11, 12].

In an effort to determine whether expression of the different Tat constructs has any effect on the propensity for Prx1 hyperoxidation a non-reducing SDS-PAGE analysis of the cell lines was conducted. The ratio of monomeric to dimeric forms of Prx1 revealed that the expression of Tat∆GFP treated with the relatively low dose of 100μM SMX-HA made the cells more susceptible to Prx1 hyperoxidation. According to the floodgate model put forth by Wood et al. [73], this outcome was expected as the R2D SDS-PAGE data showed that the Tat∆GFP cell line produced the greatest number of spots amongst the Tat-expressing cell lines. The floodgate model proposed that at low concentrations H_2_O_2_ is reduced by Prx, but as the concentration increases, Prx are inactivated by hyperoxidation thus allowing H_2_O_2_ to accumulate and oxidise other target proteins [72, 73]. The hyperoxidation of Prx1 in the Tat∆GFP-expressing cell line likely resulted in the oxidation of numerous other unique cysteine thiol target proteins to disulphides that then appeared on the R2D gels.

All eukaryotic Prx are inherently sensitive to hyperoxidation though studies have shown some are more resilient to hyperoxidation than others [80]. This is based on several factors including the rate of Prx disulphide formation, but also the local environment. For instance, in vitro*,* the ER-localized Prx4 has a similar sensitivity to hyperoxidation to cytosolic Prx1, but in vivo a negligible amount of Prx4 becomes hyperoxidized due to the low amount of disulphide reductase in the ER, which causes Prx4 disulphides to accumulate instead [46]. Both scenarios could apply in our case as the SMX-HA treatment following increasing Tat∆GFP expression resulted in a decrease in dimer formation (data not shown) suggesting the increased cytoplasmic presence of Tat∆GFP may affect the H_2_O_2_ buffering capacity of the cell due to the initial availability of reduced protein thiols. Thus, in addition to the NLS being important at the transcriptional level, the Tat NLS may ensure that the infected cell’s viability is maintained to allow viral replication to take place by minimizing Tat’s presence in the cytoplasm. Further investigation will be required to identify the unique disulphide proteins identified in cell lysates from cells infected with HIV and expressing wildtype type Tat and its mutants. It is possible that these unique disulphide proteins, especially those identified in the Tat∆GFP expressing cells following SMX-HA exposure, play a critical role in maintaining cellular redox homeostasis and cell viability.

## Conclusion

The amount of overall protein haptenation by SMX-HA/SMX-NO is independent of the presence and cellular distribution of HIV Tat. HIV infection leads to an increase in the number of mixed protein-protein disulphides (PSSP or PSSP’) present. Wildtype Tat and the mutants tested, in the absence of SMX-HA, significantly altered the disulphide proteome. The R2D SDS-PAGE experiments confirmed SMX-HA enhanced oxidative stress leading to the increased formation of mixed protein disulphides and the hyperoxidation of Prx1 in Jurkat T cells. In the presence of SMX-HA, unique disulphide proteins were identified in HIV infected and Tat mutant expressing cell lines. The cell line expressing the Tat∆GFP mutant, which accumulates in the cytoplasm, showed the most sensitivity to Prx1 hyperoxidation and inactivation, leading to the oxidation of unique target proteins that could be essential for cellular homeostasis and thus influence the complex pathogenesis of drug hypersensitivity. These findings indicate that the combination of HIV Tat and SMX-HA may alter the activity of cellular proteins required for redox homeostasis. Our previous data [21, 40] showed that HIV Tat and SMX-HA, in combination, accentuate the cytopathic effects associated with HIV infection of T cells. Inducing elevated levels of Tat72GFP in a dose-dependent manner resulted in decreased viability, increased release of cytochrome C and activation of caspase-3 suggesting apoptosis as a mechanism of cell death [9, 40]. Subsequently, using the same constructs employed in this report, we previously demonstrated reduced cell viability of cells transfected with Tat deletion mutants that were substantially present in the cytoplasm [21]. We also previously demonstrated SMX-HA results in increased ROS production via the C-terminal half of Tat [21]. Thus, when our earlier data is considered in the context of the results presented here, it enables us to propose the following model. The combined consequences of SMX-HA and an HIV Tat altered cellular redox state sets the stage for the initiation of an ADR in which Tat accentuates the release of haptenated proteins from dying cells, providing the antigens necessary for the induction of a T cell-mediated hypersensitivity reaction.

## Additional files

Additional file 1: **Figure S1.** R2D SDS-PAGE of thiol proteins, formed by reduction of cellular protein-protein mixed disulphides, in lysates of (A) Jurkat E6.1, (B) Jurkat-HIV, (C) Tat101, (D) Tat101GFP, (E) Tat72GFP and (F) Tat∆GFP at 0 ng/ml Dox. The R2D gels in panels A and B are replicas of those presented in Fig. 2. Cells from each of the various lines were treated with 0.05% DMSO for 2 h, collected and the protein was isolated. Protein lysate (85 μg) was loaded onto the first dimension gel and run for 3 h followed by an overnight run of the second dimension gel. On the left side of the diagonal on each gel are molecular weight protein standards that are enumerated to the left of panels A, C and E. (TIF 4102 kb)

Additional file 2: **Figure S2.** R2D SDS-PAGE of thiol proteins, formed by reduction of cellular protein-protein mixed disulphides, in lysates of (A) Jurkat E6.1, (B) Jurkat-HIV, (C) Tat101, (D) Tat101GFP, (E) Tat72GFP, (F) Tat∆GFP, with panels C-F induced for 40 h with 400 ng/ml Dox (C and F) and 200 ng/ml Dox (D and E) prior to DMSO treatment. The redox 2D gels in panels A and B are replicas of those represented in Fig. 2. Cells from each of the various lines were treated with 0.05% DMSO for 2 h, collected and the protein was isolated. Protein lysate (85 μg) was loaded onto the first dimension gel and run for 3 h followed by an overnight run of the second dimension gel. On the left side of the diagonal on each gel are molecular weight protein standards that are enumerated the left of panels A, C and E . (TIF 4107 kb)

Additional file 3: **Figure S3.** R2D SDS-PAGE of thiol proteins formed by reduction of cellular protein-protein mixed disulphides, in lysates of (A) Jurkat E6.1, (B) Jurkat-HIV, (C) Tat101, (D) Tat101GFP, (E) Tat72GFP and (F) Tat∆GFP at 0 ng/ml Dox. The R2D gels in panels A and B are replicas of those represented in Fig. 4. Cells from each of the various lines were treated with 200 μM SMX-HA for 2 h, collected and the protein was isolated. Protein lysate (85 μg) was loaded onto the first dimension gel and run for 3 h followed by an overnight run of the second dimension gel. On the left side of the diagonal on each gel are molecular weight protein standards that are enumerated to the left of panels A, C and E. (TIF 3759 kb)

Additional file 4: **Figure S4.** R2D SDS-PAGE of thiol proteins formed by reduction of cellular protein-protein mixed disulphides, in lysates of (A) Jurkat E6.1, (B) Jurkat-HIV, (C) Tat101, (D) Tat101GFP, (E) Tat72GFP and (F) Tat∆GFP, induced for 40 h with 400 ng/ml Dox (C and F) and 200 ng/ml Dox (D and E) prior to drug treatment. The R2D gels in panels A and B are replicas of those represented in Fig. 4. Cells from each of the various lines were then treated with 200 μM SMX-HA for 2 h, collected and the protein was isolated. Protein lysate (85 μg) was loaded onto the first dimension gel and run for 3 h followed by an overnight run of the second dimension gel. On the left side of the diagonal on each gel are molecular weight protein standards that are enumerated to the left of panels A, C and E. (TIF 3762 kb)

## Abbreviations

ADR: Adverse drug reaction
AIDS: Acquired immunodeficiency syndrome
DMSO: Dimethyl sulfoxide
Dox: Doxycycline
DTT: Dithiothreitol
GFP: Green fluorescent protein
GSH: Glutathione
HIV-1: Human immunodeficiency virus type 1
IA: Iodoacetamide
Mn-SOD: Manganese super oxide dismutase
MS: Mass spectrometry
NLS: Nuclear localization sequence
Prx: Peroxiredoxins
PSH: Protein thiol
PSSP: Protein-protein disulphide
PTD: Protein transduction domain
R2D: Redox two-dimensional
ROS: Reactive oxygen species
SH: Sulphydryl group
SMX: Sulphamethoxazole
SMX-HA: Sulphamethoxazole-hydroxylamine
SMX-NO: Sulphamethoxazole-nitroso
