# Supplementary figures and images for: HIV-1 tat expression and sulphamethoxazole hydroxylamine mediated oxidative stress alter the disulfide proteome in Jurkat T cells
Source: Virol J. 2018 May 9;15:82. doi: 10.1186/s12985-018-0991-x (PMC5944096; doi:10.1186/s12985-018-0991-x)

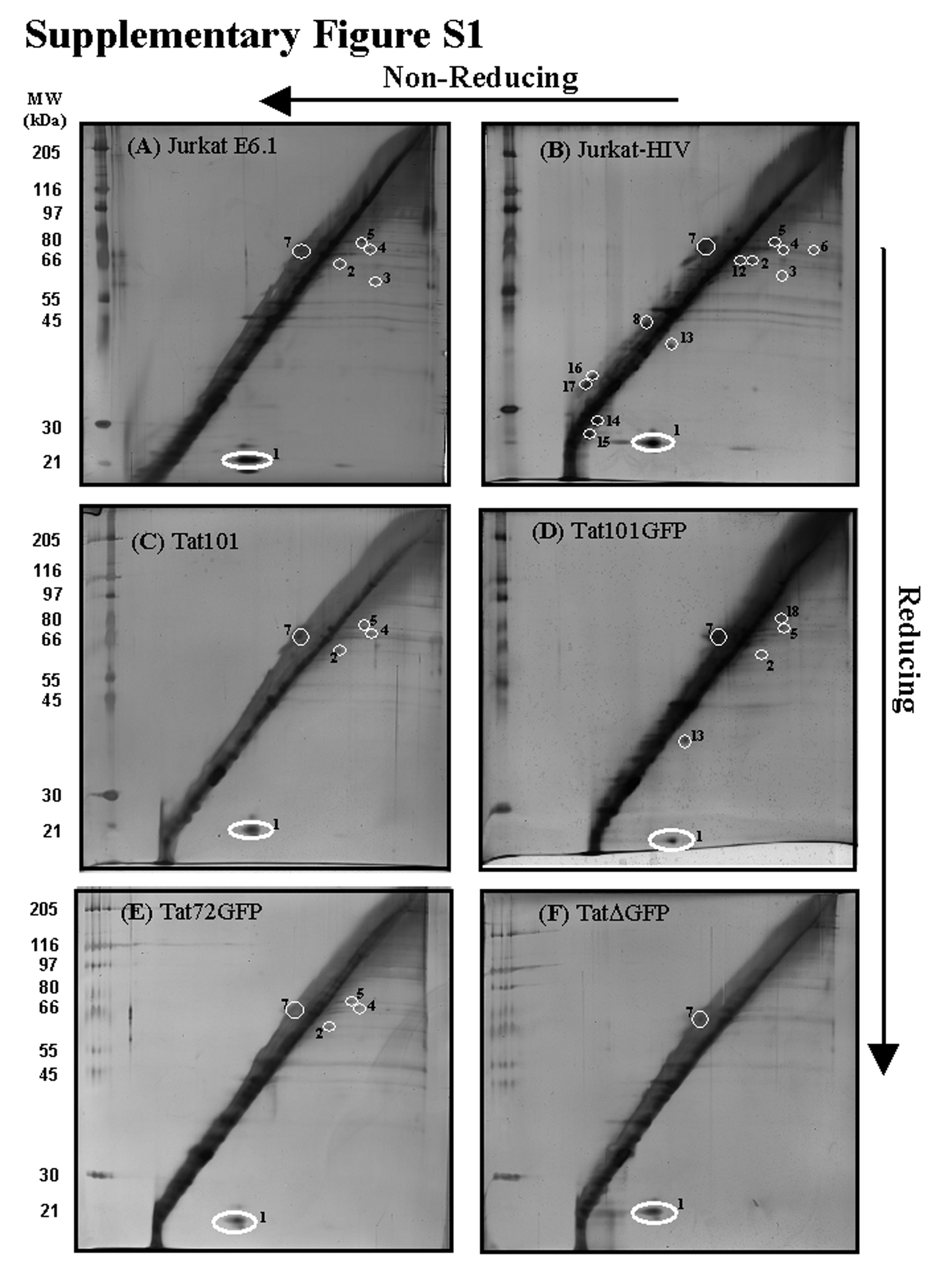

Supplement: Supplementary file 1 — Figure S1. R2D SDS-PAGE of thiol proteins, formed by reduction of cellular protein-protein mixed disulphides, in lysates of (A) Jurkat E6.1, (B) Jurkat-HIV, (C) Tat101, (D) Tat101GFP, (E) Tat72GFP and (F) Tat∆GFP at 0 ng/ml Dox. The R2D gels in panels A and B are replicas of those presented in Fig. 2. Cells from each of the various lines were treated with 0.05% DMSO for 2 h, collected and the protein was isolated. Protein lysate (85 μg) was loaded onto the first dimension gel and run for 3 h followed by an overnight run of the second dimension gel. On the left side of the diagonal on each gel are molecular weight protein standards that are enumerated to the left of panels A, C and E. (TIF 4102 kb) [file 12985_2018_991_MOESM1_ESM.tif]

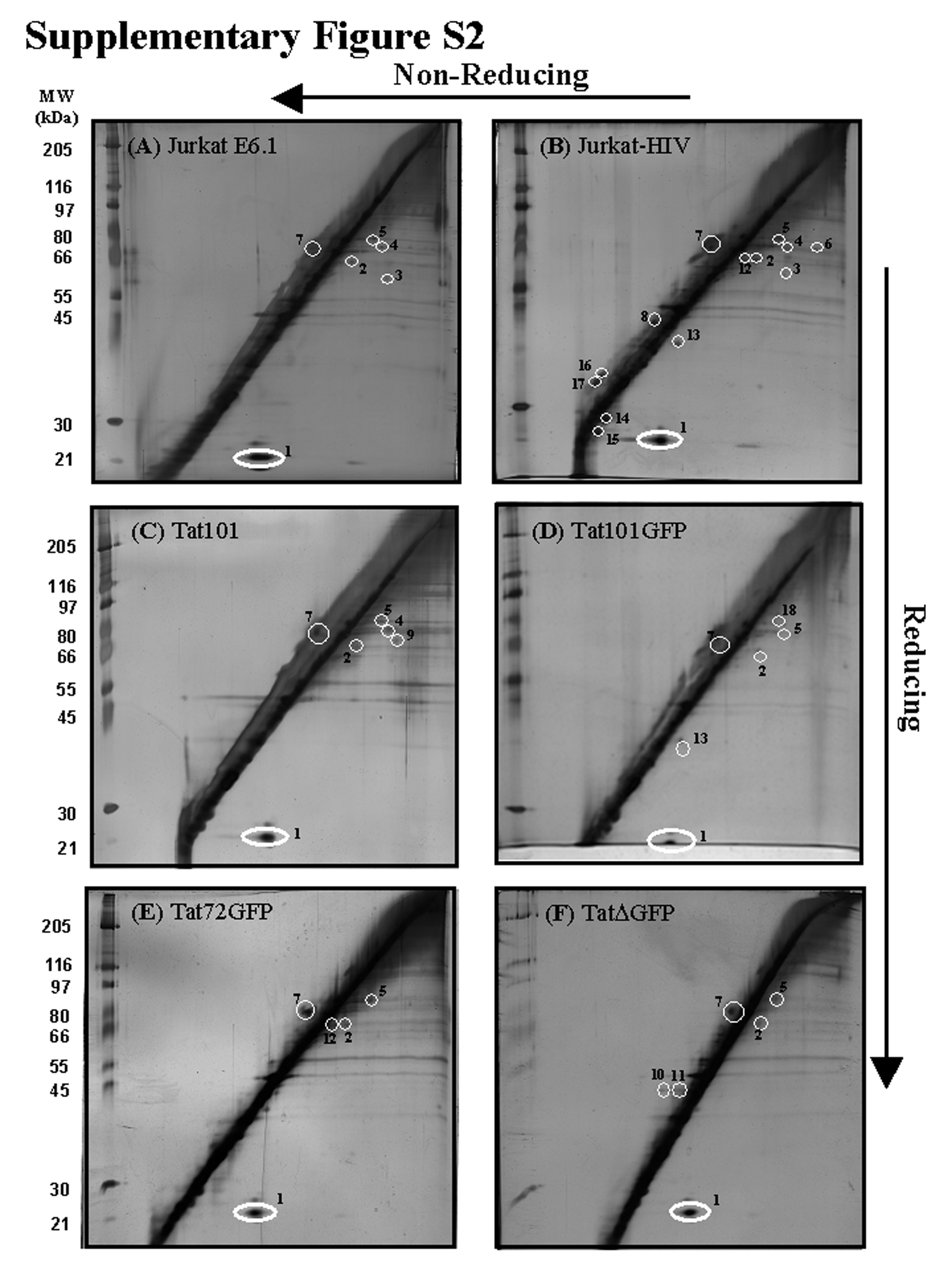

Supplement: Supplementary file 2 — Figure S2. R2D SDS-PAGE of thiol proteins, formed by reduction of cellular protein-protein mixed disulphides, in lysates of (A) Jurkat E6.1, (B) Jurkat-HIV, (C) Tat101, (D) Tat101GFP, (E) Tat72GFP, (F) Tat∆GFP, with panels C-F induced for 40 h with 400 ng/ml Dox (C and F) and 200 ng/ml Dox (D and E) prior to DMSO treatment. The redox 2D gels in panels A and B are replicas of those represented in Fig. 2. Cells from each of the various lines were treated with 0.05% DMSO for 2 h, collected and the protein was isolated. Protein lysate (85 μg) was loaded onto the first dimension gel and run for 3 h followed by an overnight run of the second dimension gel. On the left side of the diagonal on each gel are molecular weight protein standards that are enumerated the left of panels A, C and E . (TIF 4107 kb) [file 12985_2018_991_MOESM2_ESM.tif]

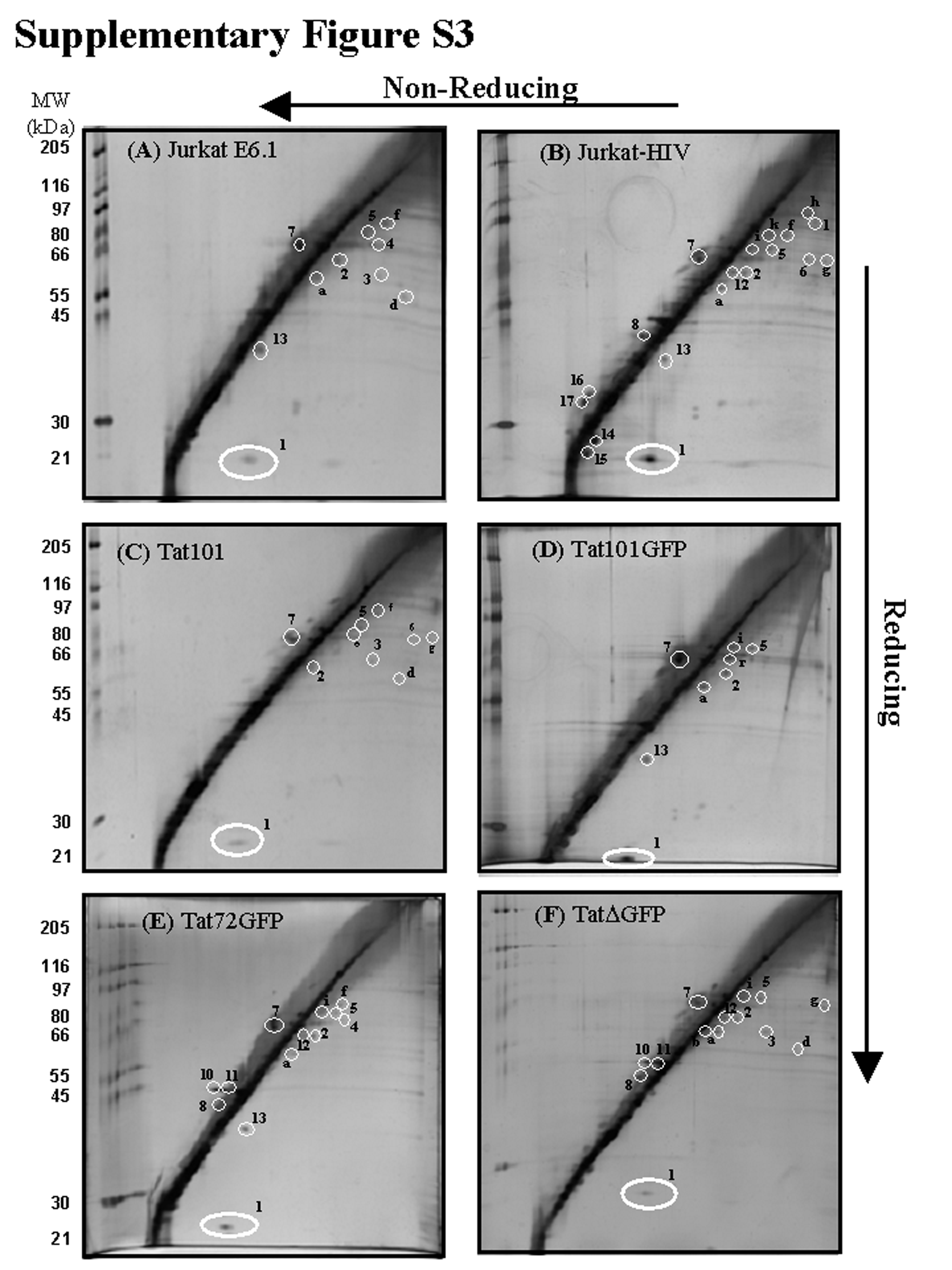

Supplement: Supplementary file 3 — Figure S3. R2D SDS-PAGE of thiol proteins formed by reduction of cellular protein-protein mixed disulphides, in lysates of (A) Jurkat E6.1, (B) Jurkat-HIV, (C) Tat101, (D) Tat101GFP, (E) Tat72GFP and (F) Tat∆GFP at 0 ng/ml Dox. The R2D gels in panels A and B are replicas of those represented in Fig. 4. Cells from each of the various lines were treated with 200 μM SMX-HA for 2 h, collected and the protein was isolated. Protein lysate (85 μg) was loaded onto the first dimension gel and run for 3 h followed by an overnight run of the second dimension gel. On the left side of the diagonal on each gel are molecular weight protein standards that are enumerated to the left of panels A, C and E. (TIF 3759 kb) [file 12985_2018_991_MOESM3_ESM.tif]

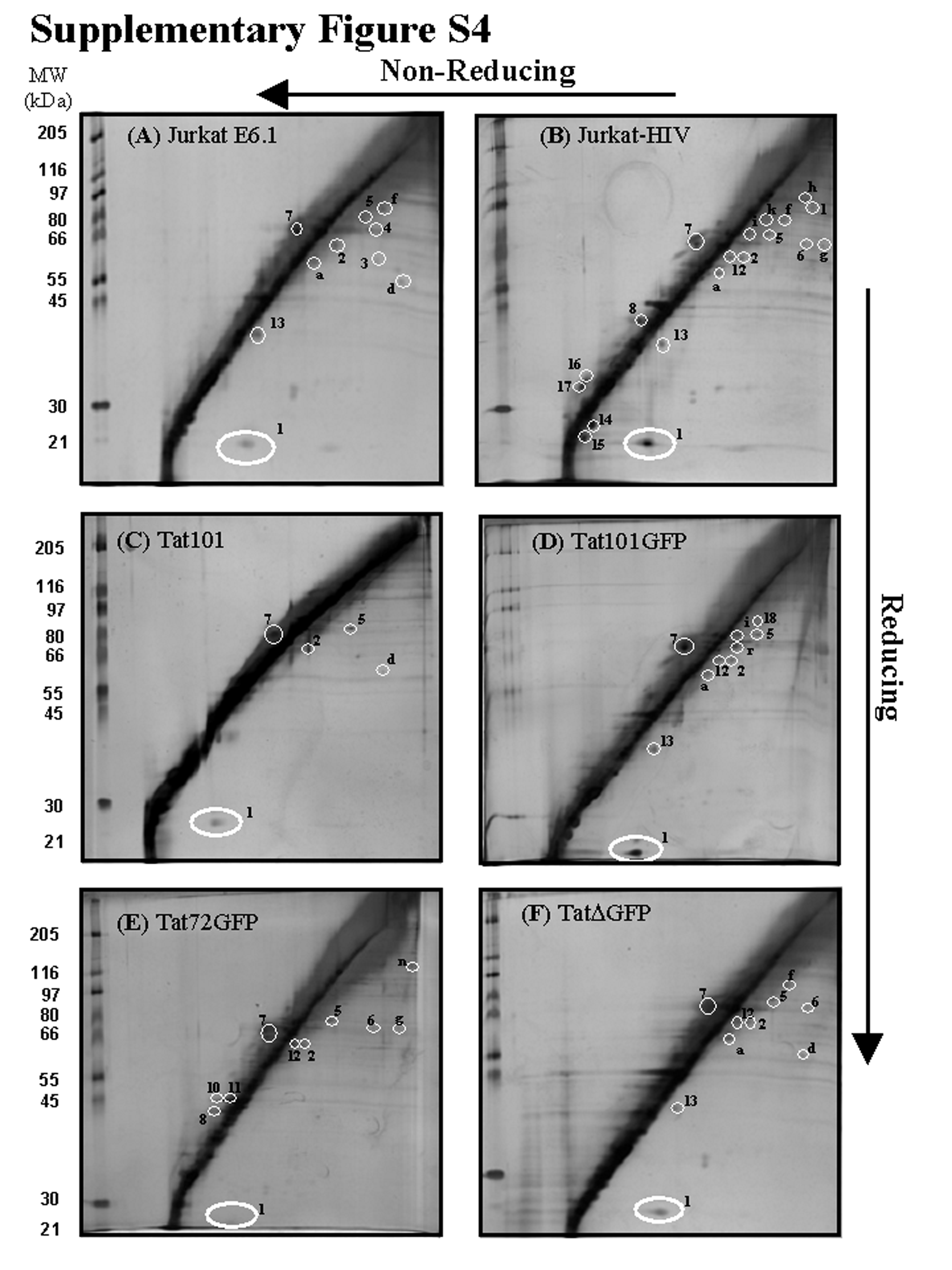

Supplement: Supplementary file 4 — Figure S4. R2D SDS-PAGE of thiol proteins formed by reduction of cellular protein-protein mixed disulphides, in lysates of (A) Jurkat E6.1, (B) Jurkat-HIV, (C) Tat101, (D) Tat101GFP, (E) Tat72GFP and (F) Tat∆GFP, induced for 40 h with 400 ng/ml Dox (C and F) and 200 ng/ml Dox (D and E) prior to drug treatment. The R2D gels in panels A and B are replicas of those represented in Fig. 4. Cells from each of the various lines were then treated with 200 μM SMX-HA for 2 h, collected and the protein was isolated. Protein lysate (85 μg) was loaded onto the first dimension gel and run for 3 h followed by an overnight run of the second dimension gel. On the left side of the diagonal on each gel are molecular weight protein standards that are enumerated to the left of panels A, C and E. (TIF 3762 kb) [file 12985_2018_991_MOESM4_ESM.tif]
